# Supplementary material for: Krüppel-like factor/specificity protein evolution in the Spiralia and the implications for cephalopod visual system novelties
Source: Proc Biol Sci. 2020 Oct 21;287(1937):20202055. doi: 10.1098/rspb.2020.2055 (PMC7661307; doi:10.1098/rspb.2020.2055)
Supplement: Electronic Supplementary File PDF [file rspb20202055supp1.pdf]

## Supplementary Tables

### Table S1. Primers used for *in situ* hybridization probes

| Gene Name | Forward Primer | Reverse Primer | Product Length | Cloned | *in situ* expression |
|---|---|---|---|---|---|
| SP6-9a | TTACGCCAGCGACATCTGTT | ATCCTGGTACGTGGCAACTG | 629 | Y | Y |
| SP6-9b | GCATCCCACGGACACTACAA | CTCACTCCTCCGCCGTATTC | 774 | Y | Y |
| SP5 | CATCATCACCCACACCCACA | CGCTGATGCCAAAGTCGATG | 984 | Y | Y |
| SP4 | AGGATAACCAGCCATCCCCT | CGCTTGCTGTTGAGCTTGTT | 1001 | Y | Y |
| KLF5 | TGTCCCTGCCCTATAGCCAT | TGCACTTGAATGGTTTGGCG | 552 | Y | Y |
| KLF10-11a | TCTTGCGGACAATAACCCCC | ACCACTGTGTGTCCGTGAAT | 547 | Y | N |
| KLF10-11b | GATGCCAACACTCATGGGGA | GCTGCTGCTGATGTGGAAAC | 663 | Y | N |
| KLF1/2/4 | TCACTCCACCCGCCTCTC | ATGGCCTATCACCGGTGT | 303 | Y | N |
| KLF15 | CCTCTCCGTTGGAACTGGAC | GGTGACCCGTAGAGCCTTTC | 518 | Y | N |

### Table S2. List of sequences used in this study for phylogenetic analysis (separate file)

**Table S3. Genomic coordinates of spiralian *SP6-9* genes**

| Species | Gene | Scaffold/Chromosome | Genomic Coordinates | Number of introns |
|---|---|---|---|---|
| *Euprymna scolopes* | SP6-9a | SRIE01026880 | 2,516,103-2,514,862 | 0 |
| | SP6-9b | SRIE01026880 | 1,433,033-1,430,910 | 0 |
| | SP6-9c | SRIE01026880 | 1,913,147-1,912,293 | 0 |
| *Architeuthis dux* | SP6-9a | VCCN01001623 | 10,409,659-10,410,717 | 1 |
| | SP6-9b | VCCN01001623 | 9,969,671-9,971,665 | 0 |
| | SP6-9c | VCCN01001623 | 10,163,890-10,649,915 | 0 |
| *Octopus bimaculoides* | SP6-9a | 48703 | 161139-162266 | 1 |
| | SP6-9b | 48703 | 865,260-867,230 | 0 |
| | SP6-9c | 48703 | 475809-478557 | 0 |
| *Octopus vulgaris* | SP6-9a | LG2 | 104,281,618-104,371,486 | 2 |
| | SP6-9b | LG2 | 103,583,419-103,585,398 | 0 |
| | SP6-9c | LG2 | 103,916,090-103,174,424 | 0 |
| *Pomacea canaliculata* | SP6-9-1 | LG1 | 26,173,872-26,175,266 | 1 |
| | SP6-9-2 | LG1 | 12,841,060-12,842,455 | 0 |
| *Crassostrea virginica* | SP6-9-1 | LG3 | 119,856-126,373 | 1 |
| | SP6-9-2 | LG3 | 141,807-141,829 | 1 |
| | Sp6-9-3 | LG3 | 5,051,295-5,057,912 | 1 |
| | SP6-9-4 | LG3 | 5,073,183-5,079,575 | 1 |
| *Capitella teleta* | SP6-9a | 201 | 294,390-294,329 | 0 |
| | SP6-9b | 305 | 229,982-230,329 | 0 |
| | SP6-9c | 201 | 270,279-273,336 | 2 |

**Table S4. Spiralian transcriptomes used with no *SP6-9* transcript identified (separate file)**

## Supplementary Figures

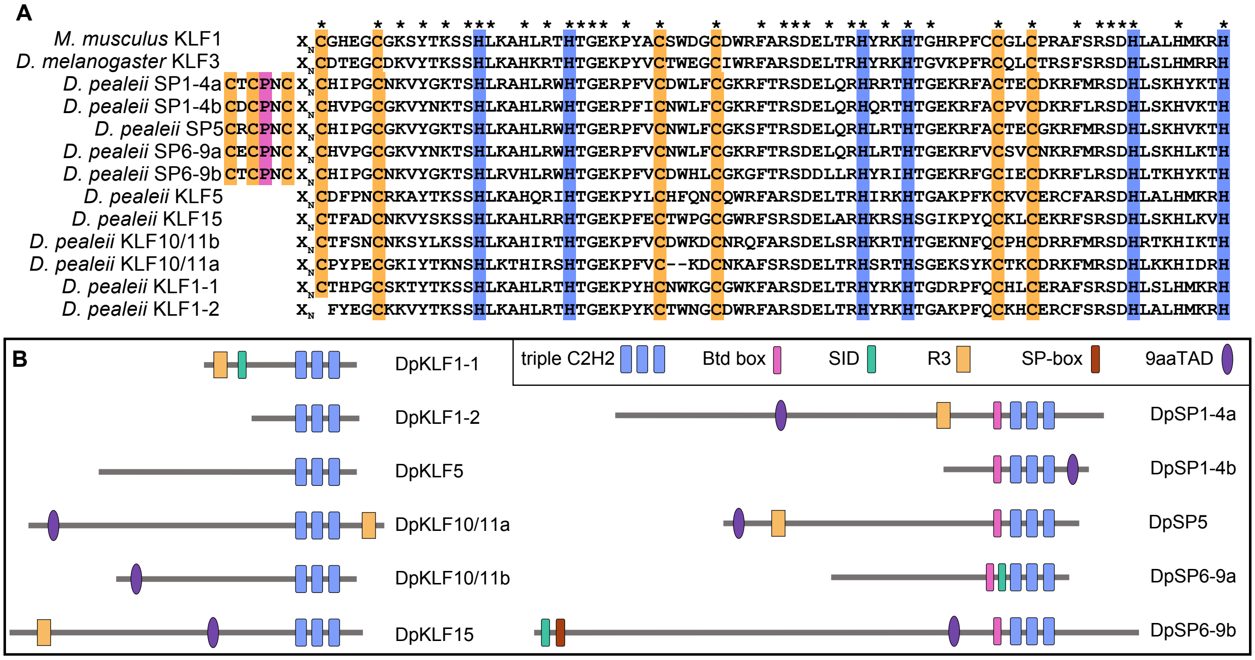


**Figure S1. Conserved functional motifs of *Doryteuthis pealeii* KLF/SP proteins. A)** The triple C2H2 zinc-finger domain of all *D. pealeii* KLF/SP proteins is highly conserved with related sequences from mouse and fruit fly. The Btd box is highly conserved in all *D. pealeii* SP proteins. Highlighted columns denote amino acids that define each domain. Asterisks indicate amino acids that are conserved across all sequences shown. **B)** *D. pealeii* KLF/SP amino acid sequences shown to scale relative to one another in gray. Locations of conserved motifs common to KLF/SPs are indicated by symbols on the sequence. Differences in sequence lengths and presence and location of motifs in the N-terminal region of the protein highlight a lack of conservation among KLF paralogs and a lack of conservation among other orthologs in the same subgroup (Presnell et al. 2015).

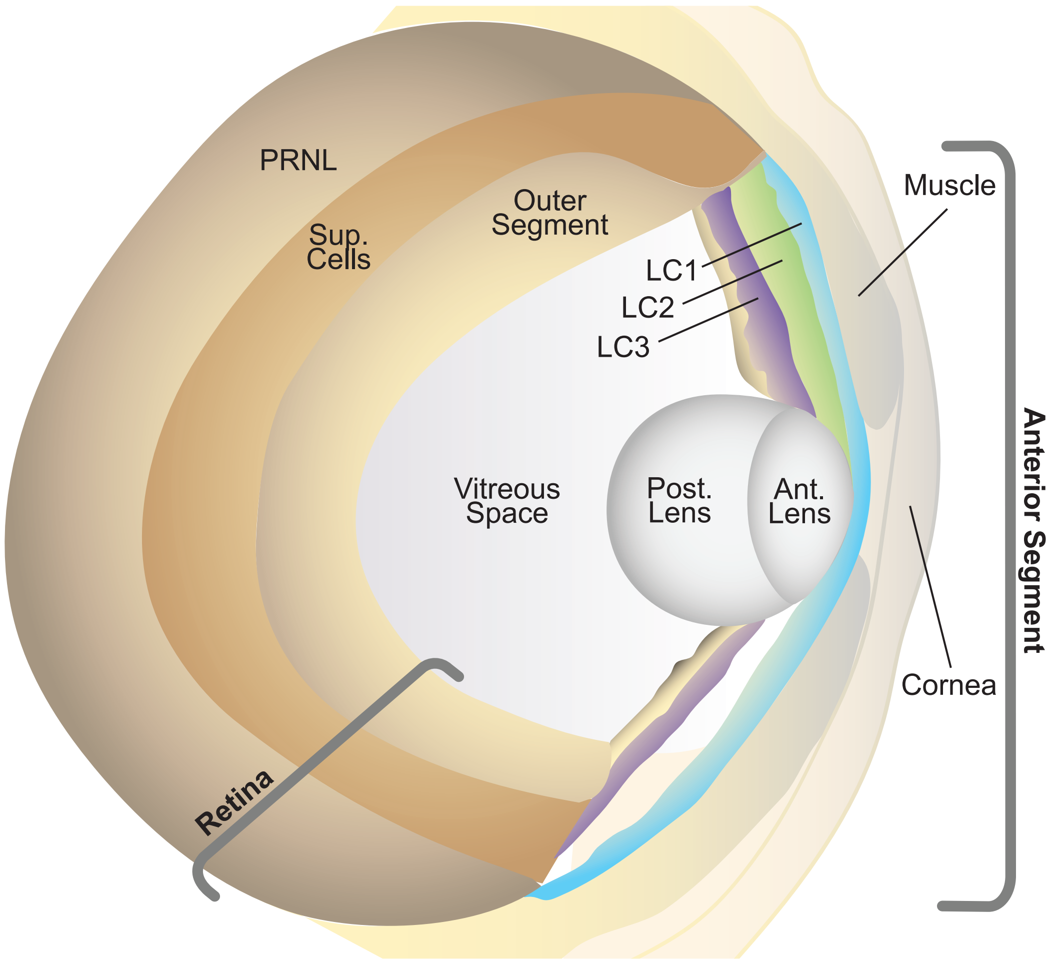


**Figure S2. Hatching stage cartoon of the *Doryteuthis pealeii* eye.** The retina is in the posterior of the eye and the lens and anterior segment is at the anterior. The retina is comprised of photoreceptor cells and support, or glial, cells (Sup. Cells). The photoreceptor cell nuclear layer is found at the posterior of the retina and the axons from these cells project directly to the optic lobe; PRNL, photoreceptor nuclear layer. The photoreceptors extend through the support cell layer and the outer segments of photoreceptor cells are at the anterior of the retina. The anterior segment is composed of three lentigentic cell populations (LC1-3), the lens, cornea and connective tissues. Distinct lentigenic cells make the posterior and anterior lens segments.

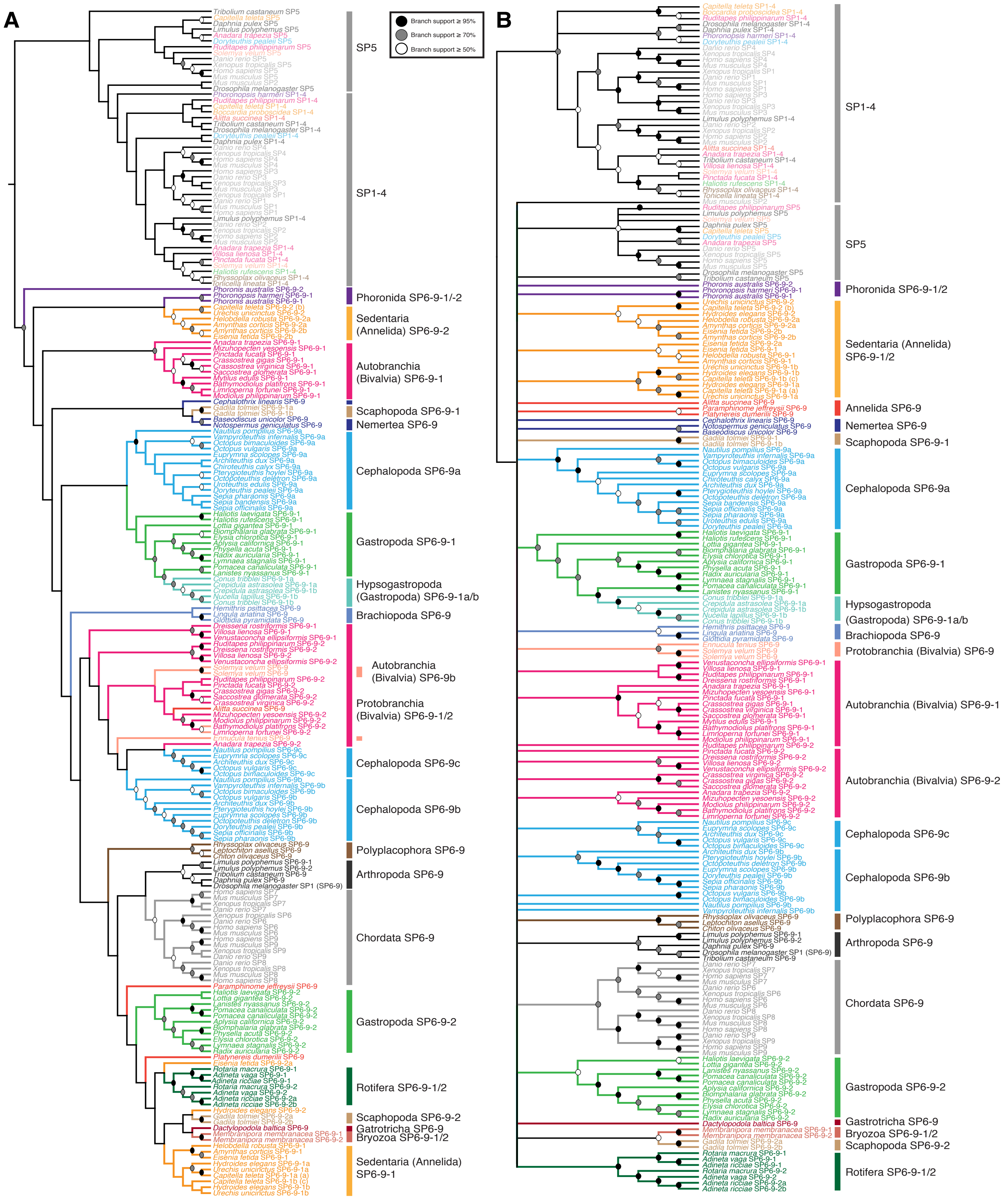

A
B
Branch support ≥ 95%
Branch support ≥ 70%
Branch support ≥ 50%
SP5
SP1-4
Phoronida SP6-9-1/-2
Sedentaria (Annelida) SP6-9-2
Autobranchia (Bivalvia) SP6-9-1
Scaphopoda SP6-9-1
Nemertea SP6-9
Cephalopoda SP6-9a
Gastropoda SP6-9-1
Hypsogastropoda (Gastropoda) SP6-9-1a/b
Brachiopoda SP6-9
Autobranchia (Bivalvia) SP6-9b
Protobranchia (Bivalvia) SP6-9-1/2
Cephalopoda SP6-9c
Cephalopoda SP6-9b
Polyplacophora SP6-9
Arthropoda SP6-9
Chordata SP6-9
Gastropoda SP6-9-2
Rotifera SP6-9-1/2
Scaphopoda SP6-9-2
Gatrotricha SP6-9
Bryozoa SP6-9-1/2
Sedentaria (Annelida) SP6-9-1
SP1-4
SP5
Phoronida SP6-9-1/2
Sedentaria (Annelida) SP6-9-1/2
Annelida SP6-9
Nemertea SP6-9
Scaphopoda SP6-9-1
Cephalopoda SP6-9a
Gastropoda SP6-9-1
Hypsogastropoda (Gastropoda) SP6-9-1a/b
Brachiopoda SP6-9
Protobranchia (Bivalvia) SP6-9
Autobranchia (Bivalvia) SP6-9-1
Autobranchia (Bivalvia) SP6-9-2
Cephalopoda SP6-9c
Cephalopoda SP6-9b
Polyplacophora SP6-9
Arthropoda SP6-9
Chordata SP6-9
Gastropoda SP6-9-2
Gastrotricha SP6-9
Bryozoa SP6-9-1/2
Scaphopoda SP6-9-2
Rotifera SP6-9-1/2

**Figure S3. Multiple SP6-9 paralogs are found in Spiralia.** A) Bayesian and B) Maximum likelihood amino acid trees support SP6-9 as a monophyletic group. Sub-clades of specific gene duplicates in Spiralia are well supported within each lineage, however support for orthology among SP6-9 duplicates in each lineage are unclear. Cephalopods have at least three SP6-9 genes, although *D. pealeii* only has two in our transcriptome. Trees are color-coded to highlight major groups within Sprialia. Shaded circles at nodes represent support for the branch leading to that node, from bootstrapping (ML tree, A) or posterior probabilities (Bayesian tree, B). Black indicates support above 95%; Gray, above 70%; White, above 50%. Nodes without circles have lower than 50% support.

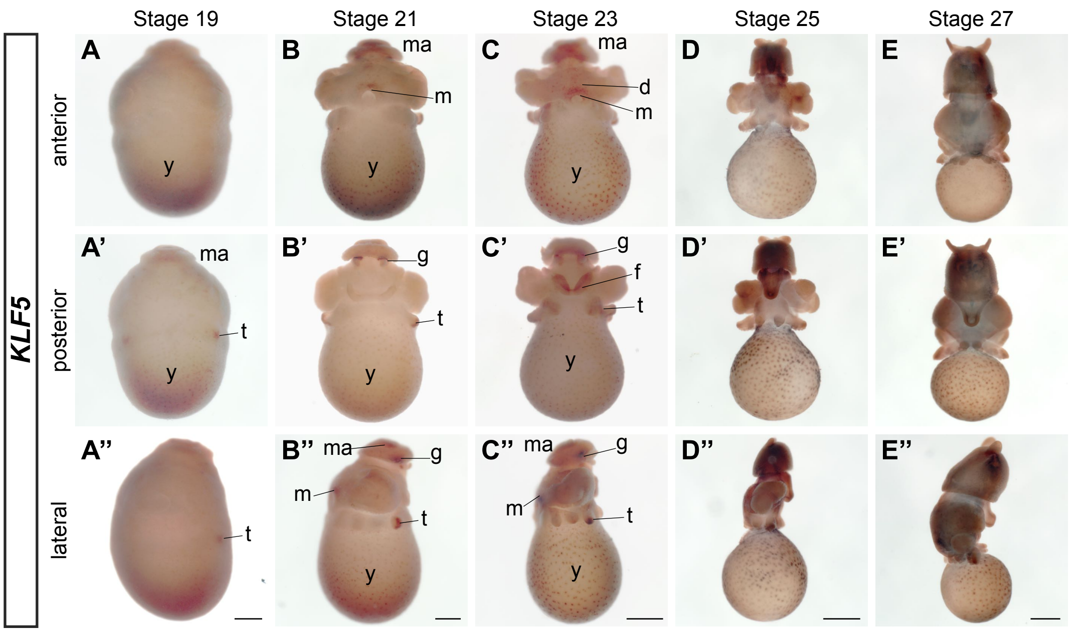


**Figure S4. *DpKLF5* expression.** Anterior (A-E), posterior (A'-E'), and lateral (A"-E") views of *DpKLF5* expression in developing embryos. *DpKLF5* is highly expressed in two arms, around the mouth, the mantle, just above the gills, in the funnel and in yolk cells. t, tentacle; d, digestive tract; f, funnel; g, gills; ma, mantle; mo, mouth; y, yolk. Scale bars, 200 μm.

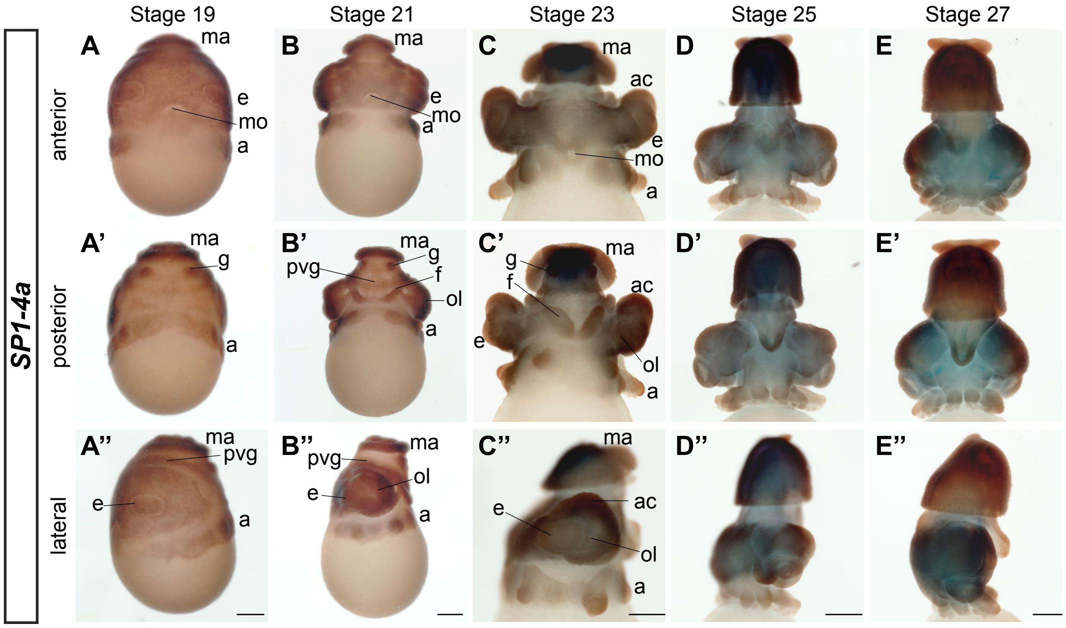


**Figure S5. *DpSP1-4a* expression resembles that of other animals.** Anterior (A-E), posterior (A'-E'), and lateral (A"-E") views of *SP1-4a* expression in developing embryos. Expression is broad and found in all developing organs, similar to arthropod and vertebrate expression patterns of *SP1-4*. This is unlike background noise, which would be present in between the developing organs and in the yolk. This expression pattern is highly reproducible. a, arms; ac, anterior chamber organ; e, eye; f, funnel; g, gills; ma, mantle; mo, mouth; ol, optic lobe; pvg, paleovisceral ganglion; y, yolk. Scale bars, 200 µm.

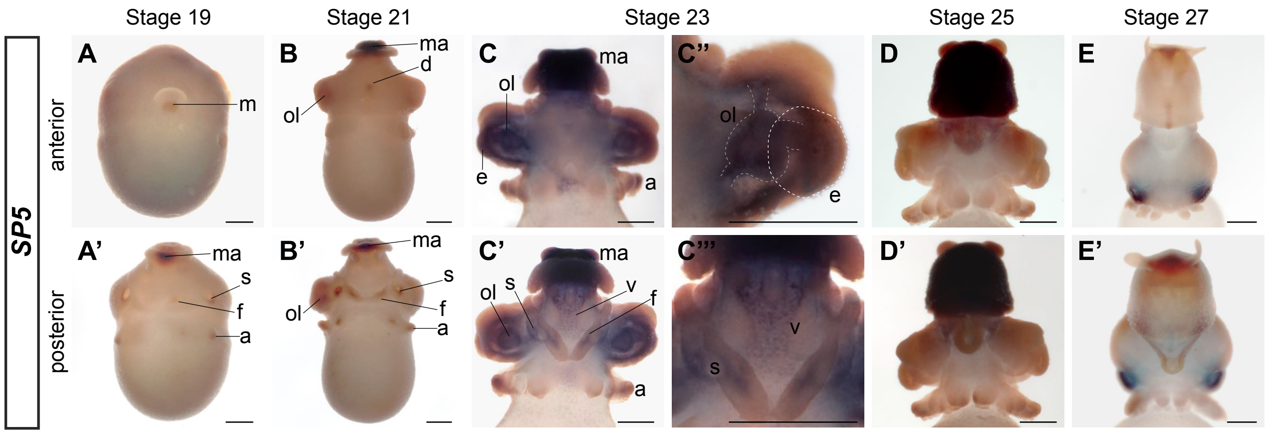


**Figure S6. *DpSP5* is expressed in sensory, neural, and circulatory structures.** Anterior (A-E) and posterior (A’-E’) views of *DpSP5* expression in the mantle, optic lobe, statocysts, funnel, gills, and tentacles. A) At stage 19, expression can be seen on the ventral side of the developing mouth. A') Posteriorly, expression is seen in the developing arms, the ventral side of the statocyst pits, and on the mantle. B,B') At stage 21, the mouth expression has moved dorsally in the gut, and the arms, statocyst and mantle expression is maintained. The first funnel and optic lobe expression is visible. C, C') At stage 23, optic lobe expression is clear and statocyst expression is maintained. C”) A closeup anterior view of the optic lobe expression at stage 23. Gray dashed line, optic lobe; white dashed line, orbit of the eye. C”’) A closeup posterior view of statocyst expression and new superficial expression on the posterior of the embryo above the funnel is detected. This appears to follow a track dorsally, splitting into two fields and surrounding the gill rudiment. This may follow the track of the future vena cava. D, D') At stage 25 the mantle is darkly stained and the statocysts are still expressing. E, E') At stage 27 the only stain visible is in the statocyst and anterior segment. *DpSP5* appears to decrease in expression in later stages, with little expression seen by stage 27. Although we do not see clear anterior segment expression until stage 27, this may indicate some later role of DpSP5 in anterior segment development. a, arms; d, digestive tract; e, eye; f, funnel; g, gills; ma, mantle; ol, optic lobe; pvg, paleovisceral ganglion; s, statocyst; v, vasculature; y, yolk. Scale bars, 200 µm.

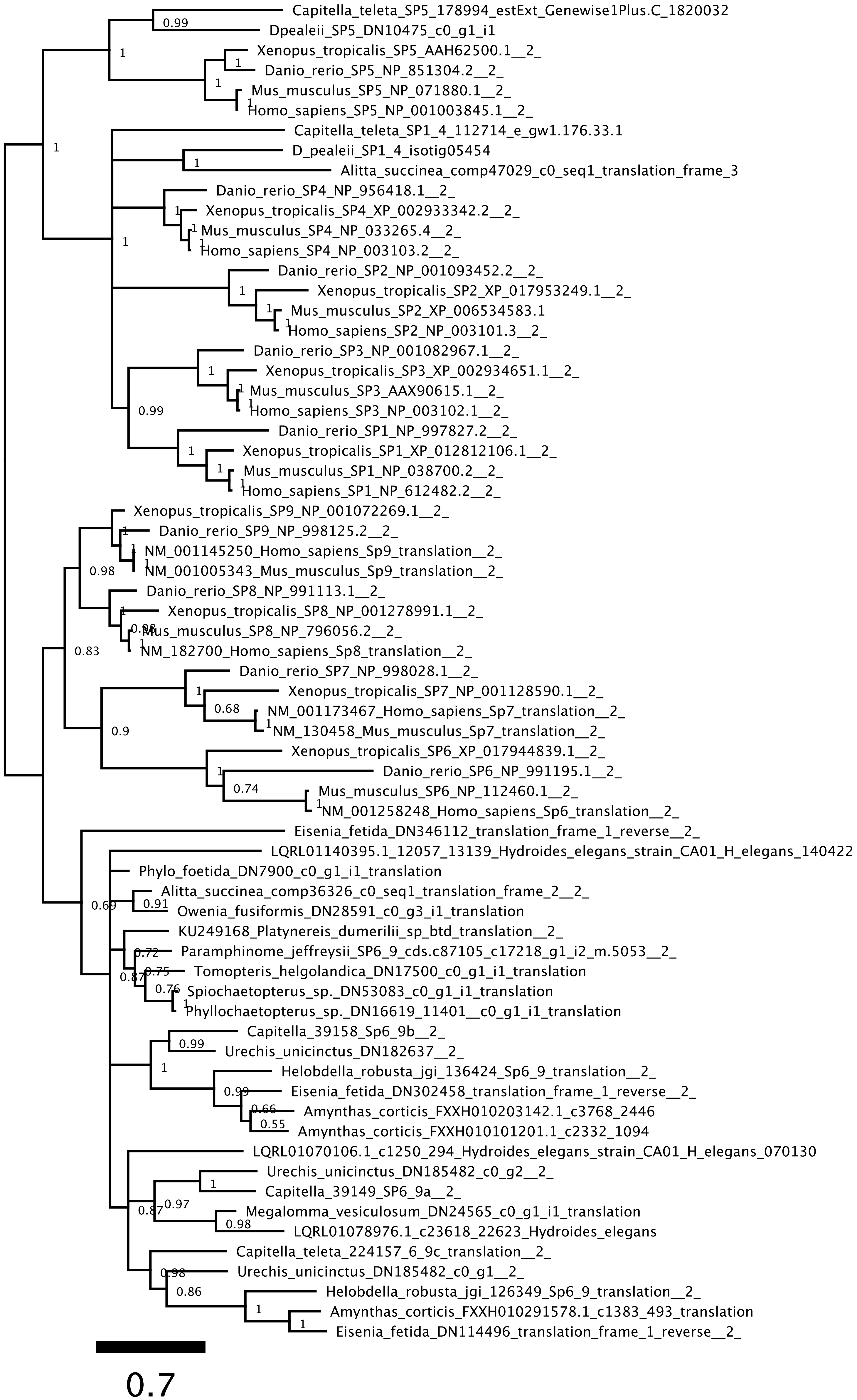

Capitella_teleta_SP5_178994_estExt_Genewise1Plus.C_1820032
Dpealeii_SP5_DN10475_c0_g1_i1
Xenopus_tropicalis_SP5_AAH62500.1__2_
Danio_rerio_SP5_NP_851304.2__2_
Mus_musculus_SP5_NP_071880.1__2_
Homo_sapiens_SP5_NP_001003845.1__2_
Capitella_teleta_SP1_4_112714_e_gw1.176.33.1
D_pealeii_SP1_4_isotig05454
Alitta_succinea_comp47029_c0_seq1_translation_frame_3
Danio_rerio_SP4_NP_956418.1__2_
Xenopus_tropicalis_SP4_XP_002933342.2__2_
Mus_musculus_SP4_NP_033265.4__2_
Homo_sapiens_SP4_NP_003103.2__2_
Danio_rerio_SP2_NP_001093452.2__2_
Xenopus_tropicalis_SP2_XP_017953249.1__2_
Mus_musculus_SP2_XP_006534583.1
Homo_sapiens_SP2_NP_003101.3__2_
Danio_rerio_SP3_NP_001082967.1__2_
Xenopus_tropicalis_SP3_XP_002934651.1__2_
Mus_musculus_SP3_AAX90615.1__2_
Homo_sapiens_SP3_NP_003102.1__2_
Danio_rerio_SP1_NP_997827.2__2_
Xenopus_tropicalis_SP1_XP_012812106.1__2_
Mus_musculus_SP1_NP_038700.2__2_
Homo_sapiens_SP1_NP_612482.2__2_
Xenopus_tropicalis_SP9_NP_001072269.1__2_
Danio_rerio_SP9_NP_998125.2__2_
NM_001145250_Homo_sapiens_Sp9_translation__2_
NM_001005343_Mus_musculus_Sp9_translation__2_
Danio_rerio_SP8_NP_991113.1__2_
Xenopus_tropicalis_SP8_NP_001278991.1__2_
Mus_musculus_SP8_NP_796056.2__2_
NM_182700_Homo_sapiens_Sp8_translation__2_
Danio_rerio_SP7_NP_998028.1__2_
Xenopus_tropicalis_SP7_NP_001128590.1__2_
NM_001173467_Homo_sapiens_Sp7_translation__2_
NM_130458_Mus_musculus_Sp7_translation__2_
Xenopus_tropicalis_SP6_XP_017944839.1__2_
Danio_rerio_SP6_NP_991195.1__2_
Mus_musculus_SP6_NP_112460.1__2_
NM_001258248_Homo_sapiens_Sp6_translation__2_
Eisenia_fetida_DN346112_translation_frame_1_reverse__2_
LQRL01140395.1_12057_13139_Hydroides_elegans_strain_CA01_H_elegans_140422
Phylo_foetida_DN7900_c0_g1_i1_translation
Alitta_succinea_comp36326_c0_seq1_translation_frame_2__2_
Owenia_fusiformis_DN28591_c0_g3_i1_translation
KU249168_Platynereis_dumerilii_sp_btd_translation__2_
Paramphinome_jeffreysii_SP6_9_cds.c87105_c17218_g1_i2_m.5053__2_
Tomopteris_helgolandica_DN17500_c0_g1_i1_translation
Spiochaetopterus_sp._DN53083_c0_g1_i1_translation
Phyllochaetopterus_sp._DN16619_11401__c0_g1_i1_translation
Capitella_39158_Sp6_9b__2_
Urechis_unicinctus_DN182637__2_
Helobdella_robusta_jgi_136424_Sp6_9_translation__2_
Eisenia_fetida_DN302458_translation_frame_1_reverse__2_
Amynthas_corticis_FXXH010203142.1_c3768_2446
Amynthas_corticis_FXXH010101201.1_c2332_1094
LQRL01070106.1_c1250_294_Hydroides_elegans_strain_CA01_H_elegans_070130
Urechis_unicinctus_DN185482_c0_g2__2_
Capitella_39149_SP6_9a__2_
Megalomma_vesiculosum_DN24565_c0_g1_i1_translation
LQRL01078976.1_c23618_22623_Hydroides_elegans
Capitella_teleta_224157_6_9c_translation__2_
Urechis_unicinctus_DN185482_c0_g1__2_
Helobdella_robusta_jgi_126349_Sp6_9_translation__2_
Amynthas_corticis_FXXH010291578.1_c1383_493_translation
Eisenia_fetida_DN114496_translation_frame_1_reverse__2_
0.7

**Figure S7. Bayesian tree of annelid SP6-9 amino acid sequences.** SP6-9 sequences were mined from available transcriptomes and genomes in Annelida, with particular focus on capturing basally branching lineages. SP1-5 and vertebrate SP6-9 sequences were used as outgroups. The branch of Sedentaria including Echiura, Capitellidae, Clitellata, and Serpulidae has three SP6-9 paralogs while all other annelids have only one. The pattern of support for specific branches of SP6-9 paralogs suggests duplication then loss, followed by another round of duplication in Clitellata. The *Eisenia* SP6-9 sequence that is an outgroup to the rest of annelid SP6-9s is divergent, and probably not reflective of actual phylogenetic history.

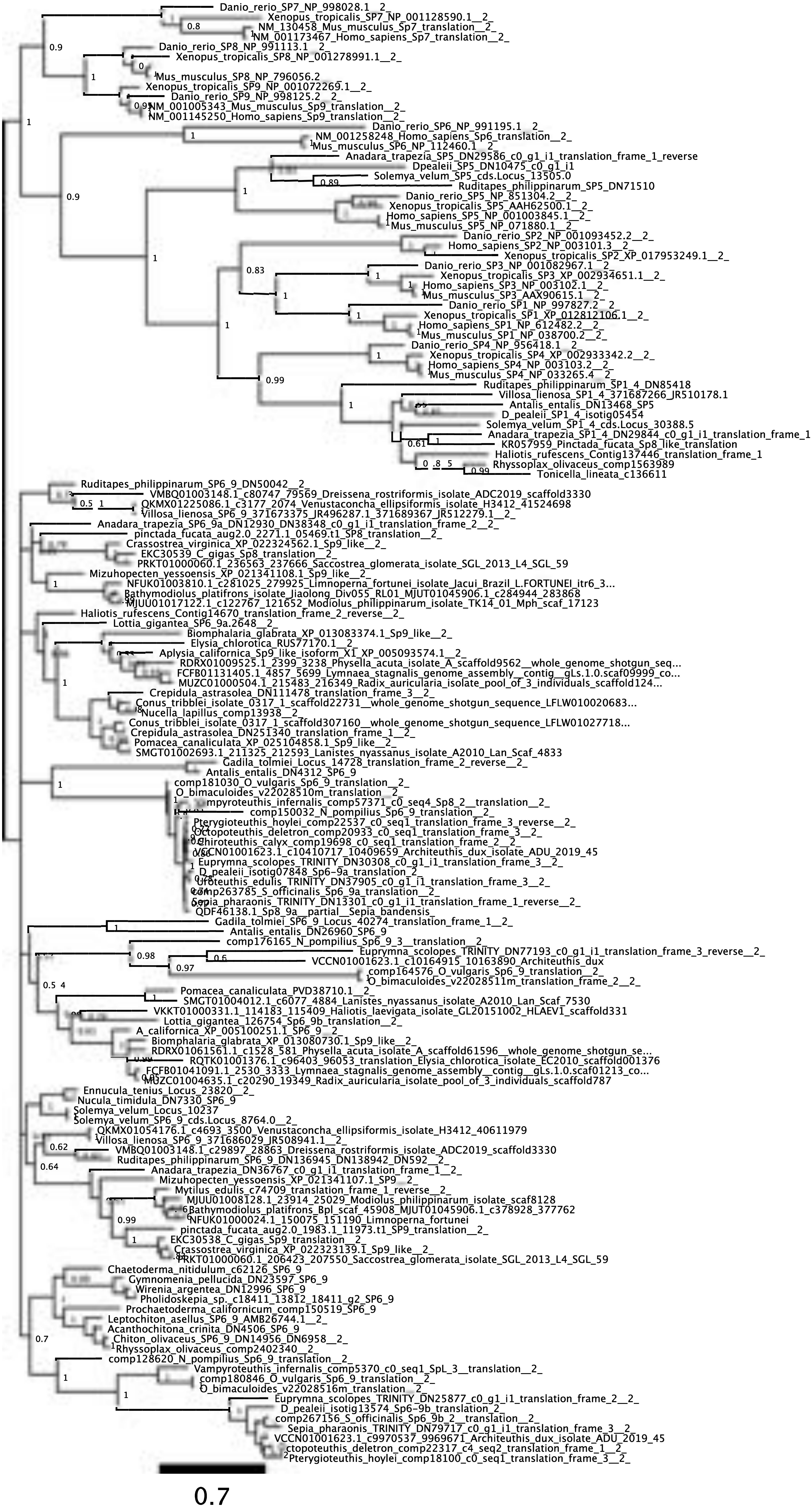

Danio_rerio_SP7_NP_998028.1__2_
Xenopus_tropicalis_SP7_NP_001128590.1__2_
NM_130458_Mus_musculus_Sp7_translation__2_
NM_001173467_Homo_sapiens_Sp7_translation__2_
Danio_rerio_SP8_NP_991113.1__2_
Xenopus_tropicalis_SP8_NP_001278991.1__2_
Mus_musculus_SP8_NP_796056.2__2_
Xenopus_tropicalis_SP9_NP_001072269.1__2_
Danio_rerio_SP9_NP_998125.2__2_
NM_001005343_Mus_musculus_Sp9_translation__2_
NM_001145250_Homo_sapiens_Sp9_translation__2_
Danio_rerio_SP6_NP_991195.1__2_
NM_001258248_Homo_sapiens_Sp6_translation__2_
Mus_musculus_SP6_NP_112460.1__2_
Anadara_trapezia_SP5_DN29586_c0_g1_i1_translation_frame_1_reverse
Dpealeii_SP5_DN10475_c0_g1_i1
Solemya_velum_SP5_cds.Locus_13505.0
Ruditapes_philippinarum_SP5_DN71510
Danio_rerio_SP5_NP_851304.2__2_
Xenopus_tropicalis_SP5_AAH62500.1__2_
Homo_sapiens_SP5_NP_001003845.1__2_
Mus_musculus_SP5_NP_071880.1__2_
Danio_rerio_SP2_NP_001093452.2__2_
Homo_sapiens_SP2_NP_003101.3__2_
Xenopus_tropicalis_SP2_XP_017953249.1__2_
Danio_rerio_SP3_NP_001082967.1__2_
Xenopus_tropicalis_SP3_XP_002934651.1__2_
Homo_sapiens_SP3_NP_003102.1__2_
Mus_musculus_SP3_AAX90615.1__2_
Danio_rerio_SP1_NP_997827.2__2_
Xenopus_tropicalis_SP1_XP_012812106.1__2_
Homo_sapiens_SP1_NP_612482.2__2_
Mus_musculus_SP1_NP_038700.2__2_
Danio_rerio_SP4_NP_956418.1__2_
Xenopus_tropicalis_SP4_XP_002933342.2__2_
Homo_sapiens_SP4_NP_003103.2__2_
Mus_musculus_SP4_NP_033265.4__2_
Ruditapes_philippinarum_SP1_4_DN85418
Villosa_lienosa_SP1_4_371687266_JR510178.1
Antalis_entalis_DN13468_SP5
D_pealeii_SP1_4_isotig05454
Solemya_velum_SP1_4_cds.Locus_30388.5
Anadara_trapezia_SP1_4_DN29844_c0_g1_i1_translation_frame_1
KR057959_Pinctada_fucata_Sp8_like_translation
Haliotis_rufescens_Contig137446_translation_frame_1
Rhyssoplax_olivaceus_comp1563989
Tonicella_lineata_c136611
Ruditapes_philippinarum_SP6_9_DN50042__2_
VMBQ01003148.1_c80747_79569_Dreissena_rostriformis_isolate_ADC2019_scaffold3330
QKMX01225086.1_c3177_2074_Venustaconcha_ellipsiformis_isolate_H3412_41524698
Villosa_lienosa_SP6_9_371673375_JR496287.1_371689367_JR512279.1__2_
Anadara_trapezia_SP6_9a_DN12930_DN38348_c0_g1_i1_translation_frame_2__2_
pinctada_fucata_aug2.0_2271.1_05469.t1_SP8_translation__2_
Crassostrea_virginica_XP_022324562.1_Sp9_like__2_
EKC30539_C_gigas_Sp8_translation__2_
PRKT01000060.1_236563_237666_Saccostrea_glomerata_isolate_SGL_2013_L4_SGL_59
Mizuhopecten_yessoensis_XP_021341108.1_Sp9_like__2_
NFUK01003810.1_c281025_279925_Limnoperna_fortunei_isolate_Jacui_Brazil_L.FORTUNEI_itr6_3...
Bathymodiolus_platifrons_isolate_Jiaolong_Div055_RL01_MJUT01045906.1_c284944_283868
MJUU01017122.1_c122767_121652_Modiolus_philippinarum_isolate_TK14_01_Mph_scaf_17123
Haliotis_rufescens_Contig14670_translation_frame_2_reverse__2_
Lottia_gigantea_SP6_9a.2648__2_
Biomphalaria_glabrata_XP_013083374.1_Sp9_like__2_
Elysia_chlorotica_RUS77170.1__2_
Aplysia_californica_Sp9_like_isoform_X1_XP_005093574.1__2_
RDRX01009525.1_2399_3238_Physella_acuta_isolate_A_scaffold9562__whole_genome_shotgun_seq...
FCFB01131405.1_4857_5699_Lymnaea_stagnalis_genome_assembly__contig__gLs.1.0.scaf09999_co...
MUZC01000504.1_215483_216349_Radix_auricularia_isolate_pool_of_3_individuals_scaffold124...
Crepidula_astrasolea_DN111478_translation_frame_3__2_
Conus_tribblei_isolate_0317_1_scaffold22731__whole_genome_shotgun_sequence_LFLW010020683...
Nucella_lapillus_comp13938__2_
Conus_tribblei_isolate_0317_1_scaffold307160__whole_genome_shotgun_sequence_LFLW01027718...
Crepidula_astrasolea_DN251340_translation_frame_1__2_
Pomacea_canaliculata_XP_025104858.1_Sp9_like__2_
SMGT01002693.1_211325_212593_Lanistes_nyassanus_isolate_A2010_Lan_Scaf_4833
Gadila_tolmiei_Locus_14728_translation_frame_2_reverse__2_
Antalis_entalis_DN4312_SP6_9
comp181030_O_vulgaris_Sp6_9_translation__2_
O_bimaculoides_v22028510m_translation__2_
mpyroteuthis_infernalis_comp57371_c0_seq4_Sp8_2__translation__2_
comp150032_N_pompilius_Sp6_9_translation__2_
Pterygioteuthis_hoylei_comp22537_c0_seq1_translation_frame_3_reverse__2_
Octopoteuthis_deletron_comp20933_c0_seq1_translation_frame_3__2_
Chiroteuthis_calyx_comp19698_c0_seq1_translation_frame_2__2_
VCCN01001623.1_c10410717_10409659_Architeuthis_dux_isolate_ADU_2019_45
Euprymna_scolopes_TRINITY_DN30308_c0_g1_i1_translation_frame_3__2_
D_pealeii_isotig07848_Sp6-9a_translation__2_
Uroteuthis_edulis_TRINITY_DN37905_c0_g1_i1_translation_frame_3__2_
comp263785_S_officinalis_Sp6_9a_translation__2_
Sepia_pharaonis_TRINITY_DN13301_c0_g1_i1_translation_frame_1_reverse__2_
QDF46138.1_Sp8_9a__partial__Sepia_bandensis_
Gadila_tolmiei_SP6_9_Locus_40274_translation_frame_1__2_
Antalis_entalis_DN26960_SP6_9
comp176165_N_pompilius_Sp6_9_3__translation__2_
Euprymna_scolopes_TRINITY_DN77193_c0_g1_i1_translation_frame_3_reverse__2_
VCCN01001623.1_c10164915_10163890_Architeuthis_dux
comp164576_O_vulgaris_Sp6_9_translation__2_
O_bimaculoides_v22028511m_translation_frame_2__2_
Pomacea_canaliculata_PVD38710.1__2_
SMGT01004012.1_c6077_4884_Lanistes_nyassanus_isolate_A2010_Lan_Scaf_7530
VKKT01000331.1_114183_115409_Haliotis_laevigata_isolate_GL20151002_HLAEV1_scaffold331
Lottia_gigantea_126754_Sp6_9b_translation__2_
A_californica_XP_005100251.1_SP6_9__2_
Biomphalaria_glabrata_XP_013080730.1_Sp9_like__2_
RDRX01061561.1_c1528_581_Physella_acuta_isolate_A_scaffold61596__whole_genome_shotgun_se...
RQTK01001376.1_c96403_96053_translation_Elysia_chlorotica_isolate_EC2010_scaffold001376
FCFB01041091.1_2530_3333_Lymnaea_stagnalis_genome_assembly__contig__gLs.1.0.scaf01213_co...
MUZC01004635.1_c20290_19349_Radix_auricularia_isolate_pool_of_3_individuals_scaffold787
Ennucula_tenius_Locus_23820__2_
Nucula_timidula_DN7330_SP6_9
Solemya_velum_Locus_10237
Solemya_velum_SP6_9_cds.Locus_8764.0__2_
QKMX01054176.1_c4693_3500_Venustaconcha_ellipsiformis_isolate_H3412_40611979
Villosa_lienosa_SP6_9_371686029_JR508941.1__2_
VMBQ01003148.1_c29897_28863_Dreissena_rostriformis_isolate_ADC2019_scaffold3330
Ruditapes_philippinarum_SP6_9_DN136945_DN138942_DN592__2_
Anadara_trapezia_DN36767_c0_g1_i1_translation_frame_1__2_
Mizuhopecten_yessoensis_XP_021341107.1_SP9__2_
Mytilus_edulis_c74709_translation_frame_1_reverse__2_
MJUU01008128.1_23914_25029_Modiolus_philippinarum_isolate_scaf8128
Bathymodiolus_platifrons_Bpl_scaf_45908_MJUT01045906.1_c378928_377762
NFUK01000024.1_150075_151190_Limnoperna_fortunei
pinctada_fucata_aug2.0_1983.1_11973.t1_SP9_translation__2_
EKC30538_C_gigas_Sp9_translation__2_
Crassostrea_virginica_XP_022323139.1_Sp9_like__2_
PRKT01000060.1_206423_207550_Saccostrea_glomerata_isolate_SGL_2013_L4_SGL_59
Chaetoderma_nitidulum_c62126_SP6_9
Gymnomenia_pellucida_DN23597_SP6_9
Wirenia_argentea_DN12996_SP6_9
Pholidoskepia_sp._c18411_13812_18411_g2_SP6_9
Prochaetoderma_californicum_comp150519_SP6_9
Leptochiton_asellus_SP6_9_AMB26744.1__2_
Acanthochitona_crinita_DN4506_SP6_9
Chiton_olivaceus_SP6_9_DN14956_DN6958__2_
Rhyssoplax_olivaceus_comp2402340__2_
comp128620_N_pompilius_Sp6_9_translation__2_
Vampyroteuthis_infernalis_comp5370_c0_seq1_SpL_3__translation__2_
comp180846_O_vulgaris_Sp6_9_translation__2_
O_bimaculoides_v22028516m_translation__2_
Euprymna_scolopes_TRINITY_DN25877_c0_g1_i1_translation_frame_2__2_
D_pealeii_isotig13574_Sp6-9b_translation__2_
comp267156_S_officinalis_Sp6_9b_2__translation__2_
Sepia_pharaonis_TRINITY_DN79717_c0_g1_i1_translation_frame_3__2_
VCCN01001623.1_c9970537_9969671_Architeuthis_dux_isolate_ADU_2019_45
ctopoteuthis_deletron_comp22317_c4_seq2_translation_frame_1__2_
Pterygioteuthis_hoylei_comp18100_c0_seq1_translation_frame_3__2_
0.7

**Figure S8. Bayesian tree of mollusc SP6-9 amino acid sequences.** SP6-9 sequences were mined from available transcriptomes and genomes in Mollusca, with particular focus on capturing aculiferan lineages. Other SP1-5 and vertebrate SP6-9 sequences were used as outgroups. Deeper nodes have little support, similar to our larger spiralian SP6-9 trees. Additional aculiferan representatives only had a single paralog if any SP6-9 sequence was found at all.

**Separate Supplementary Data Files**

**Supplementary Data 1. Alignment file for RAxML and MrBayes tree of KLF/SP conserved domains across Metazoa**

**Supplementary Data 2. Nexus tree file for RAxML tree of KLF/SP conserved domains across Metazoa**

**Supplementary Data 3. Nexus tree file for MrBayes tree of KLF/SP conserved domains across Metazoa**

**Supplementary Data 4. Alignment file for RAxML and MrBayes tree of SP6-9 full length sequences across Spiralia**

**Supplementary Data 5. Nexus tree file for RAxML tree of SP6-9 full length sequences across Spiralia**

**Supplementary Data 6. Nexus tree file for MrBayes tree of SP6-9 full length sequences across Spiralia**

**Supplementary Data 7. Alignment file for MrBayes tree of SP6-9 full length sequences across Cephalopoda**

**Supplementary Data 8. Nexus tree file for MrBayes tree of SP6-9 full length sequences across Cephalopoda**

**Supplementary Data 9. Alignment file for MrBayes tree of SP6-9 full length sequences across Annelida**

**Supplementary Data 10. Nexus tree file for MrBayes tree of SP6-9 full length sequences across Annelida**

**Supplementary Data 11. Alignment file for MrBayes tree of SP6-9 full length sequences across Mollusca**

**Supplementary Data 12. Nexus tree file for MrBayes tree of SP6-9 full length sequences across Mollusca**
